# Effects of a 12-Week CrossFit-Adapted Program on Balance, Functional Mobility, and Lower-Limb Power in Community-Dwelling Older Adults: A Randomized Controlled Trial

**DOI:** 10.3390/healthcare13243294

**Published:** 2025-12-15

**Authors:** Lamiae El-Hajjami Nachit, Felipe León-Morillas, Marco Bergamin, Stefano Gobbo, Elif Durgut, David Cruz-Díaz

**Affiliations:** 1 Department of Health Sciences, Faculty of Health Sciences, University of Jaén, 23071 Jaén, Spain; len00002@red.ujaen.es (L.E.-H.N.); dcruz@ujaen.es (D.C.-D.); 2 Department of Nursing, Pharmacology and Physical Therapy, Faculty of Medicine and Nursing, University of Córdoba, 14004 Córdoba, Spain; 3 Department of Medicine, University of Padova, Via Giustiniani, 2, 35128 Padova, Italy; marco.bergamin@unipd.it (M.B.); stefano.gobbo@unipd.it (S.G.); 4 Department of Physiotherapy and Rehabilitation, Faculty of Health Sciences, Bezmialem Vakif University, 34055 Istanbul, Turkey; edurgut@bezmialem.edu.tr

**Keywords:** CrossFit, balance, power, older adults, randomized controlled trial

## Abstract

**Highlights:**

**What are the main findings?**

- A CrossFit-adapted program can improve functional capacity, balance, and strength in older adults.

**What is the implication of the main finding?**

- CrossFit could be an innovative alternative for older adults.

**Abstract:**

Background: CrossFit could be an innovative alternative for older adults. Traditional strength training is well-established for safety and progressive overload, while concerns exist about overexertion or poor technique in modified CrossFit, especially for those with musculoskeletal or cardiovascular conditions. However, scaled and supervised CrossFit sessions have shown low injury rates and high satisfaction among older adults. Objective: to evaluate the effects of a CrossFit-adapted program on balance and muscular power. Methods: 60 older adults participated in the study. Participants were randomized into two groups: CrossFit-adapted and control. Functional capacity, balance and strength variables were analyzed. The sample size was calculated a priori using G*Power 3.1 software, considering an effect size of 0.25 [medium], α = 0.05, and a power [1–β] of 0.80 for a repeated-measures ANOVA with two groups and three measurement points. Data were analyzed using SPSS Statistics version 25. Results: Significant improvements in balance scores were observed in the CrossFit group compared to the control group. In the Timed Up and Go test, the CrossFit group improved from 9.83 ± 1.3 s to 8.74 ± 1.1 s, [*p* = 0.002]. Lower limb muscle power increased significantly in CrossFit group across all tests: chair stand test, the stair ascent and stair descent [*p* < 0.001]. Conclusions: A CrossFit-adapted program can significantly improve functional capacity, balance, and strength in older adults.

## 1. Introduction

Conventional training programs often lack engagement and adaptability for diverse functional levels, limiting adherence among older adults [1]. CrossFit-adapted, an innovative alternative, scales high-intensity functional movements to older adults’ abilities, emphasizing multi-joint exercises that mimic daily activities [2,3]. Recent evidence suggests that such programs may elicit comparable or even superior adaptations in strength, balance, and mobility compared to conventional resistance training [4,5]. In a study by Fisher J et al. [4], male and female participants performed brief, infrequent, high-intensity resistance exercise with controlled repetition duration for 12 and 19 weeks. Significant strength gains were observed in all exercises [4]. Furthermore, the group-based and competitive nature of CrossFit may promote social interaction and motivation, overcoming a lack of interest in maintaining exercise. Additionally, CrossFit can help address psychological barriers such as fear of injury [6,7].

From a physiological standpoint, both traditional strength training and modified CrossFit target neuromuscular adaptation. However, CrossFit’s high-intensity, dynamic movements and aerobic integration may provide added cardiometabolic benefits [3], including improved VO_2_max, glycemic control, and lipid profiles, while maintaining or enhancing strength [8,9]. In contrast, traditional strength training focuses on maximal strength, with less emphasis on metabolic conditioning, limiting its broader health benefits [10].

Despite the growing popularity of high-intensity functional training in older populations, scientific evidence regarding CrossFit-based or HIFT-style interventions in this demographic remains limited and heterogeneous [11,12]. Most available studies in community-dwelling older adults have examined general functional or multicomponent training programs, rather than CrossFit-adapted protocols specifically designed for this population [12,13]. Moreover, previous research has predominantly addressed global indicators of physical function, while task-specific outcomes such as balance, gait stability, and lower-limb muscular power, which are key determinants of autonomy and fall risk, have received little attention.

Furthermore, although CrossFit is often perceived as a demanding exercise modality, the scalability and feasibility of its supervised adaptations for older adults have not been sufficiently explored. The literature remains inconclusive regarding the safety profile, adherence rates, and functional impact of CrossFit-based interventions in this population [14,15].

The global increase in the aging population reinforces the need for effective, sustainable, and appealing exercise interventions for older adults [World Health Organization, 2024] [16]. While conventional resistance training remains the gold standard for mitigating age-related functional decline, CrossFit-adapted training represents a novel and integrative alternative that simultaneously targets strength, balance, coordination, and endurance within a single structured program. This multidimensional approach could promote functional independence, reduce the incidence of falls, and contribute to improved overall quality of life in older adults.

Accordingly, the present randomized controlled trial provides novel and methodologically robust evidence by implementing a twelve-week supervised CrossFit-adapted program tailored to the physical capacities of community-dwelling older adults. The study aims to evaluate the effects of this intervention on validated indicators of balance, functional mobility, and lower-limb muscular power, thereby addressing a current gap in the literature and contributing to the understanding of the feasibility and effectiveness of applying high-intensity functional principles in older populations.

## 2. Materials and Methods

### 2.1. Study Design

This prospective study was conducted in accordance with the ethical principles of the Declaration of Helsinki and was registered at ClinicalTrials.gov [Identifier: NCT07199114]. Participants were recruited from the Geriatric and Rehabilitation Unit of the University Hospital of Jaén [Andalusia, Spain]. Baseline variables were obtained from two sources. Sociodemographic and anthropometric characteristics [age, sex, height, weight, and body mass index] were extracted from the hospital’s clinical and research database under prior ethical approval and written informed consent. In contrast, cognitive screening [Mini-Mental State Examination], self-reported physical activity, handgrip strength, and all physical function assessments described below were collected directly from the participants at baseline by trained physiotherapists following standardized protocols. A complete itemized list of all baseline variables and their data source is provided in Table A1 in Appendix A.

Before randomization, all older adults underwent a standardized medical and physical examination to ensure clinical stability, safe participation, and comparability across groups.

Inclusion criteria were: [1] ability to walk independently without assistive devices; [2] age between 60 and 80 years old; [3] community-dwelling status; and [4] preserved cognitive function sufficient to understand and follow the training protocol.

Exclusion criteria were: the presence of neuromuscular, cardiovascular, or orthopedic conditions that could compromise safety during exercise testing or training; uncontrolled hypertension; recent hospitalization [<3 months]; or any contraindication established by the medical team.

### 2.2. Outcome Measures

#### 2.2.1. Baseline Descriptive Measures

Demographic, Anthropometric and Screening Measures

Demographic variables included sex [male/female] and age [years]. Anthropometric measures included height [cm], weight [kg] and body mass index [BMI, kg/m^2^]. Cognitive status was assessed using the Mini-Mental State Examination [MMSE] to ensure adequate cognitive capacity to follow the intervention safely; this variable was used for screening purposes. These measures were collected to characterize the sample and confirm comparability between groups at baseline.

Physical Activity

Physical activity level [hours per week] was self-reported by participants using a standardized questionnaire. Participants also indicated whether they met the World Health Organization recommendation of at least 150 min of moderate-intensity physical activity per week [17].

Handgrip Strength

Handgrip strength [kg] was assessed using a digital hand dynamometer. Two attempts were performed using the dominant hand, and the best value was recorded. Handgrip strength is widely recognized as an indicator of global physical performance and overall health status in older adults.

#### 2.2.2. Primary Outcome Measures

**Balance Assessment**

Balance performance was examined through a combination of static and dynamic tests to obtain a multidimensional profile of postural control.

Static balance was assessed using the Romberg test [ROM, seconds], which measures the ability to maintain a stable upright posture with eyes closed and feet together. This test is widely recognized as a reliable indicator of postural stability and sensory integration in older adults [18,19].

Dynamic balance and functional mobility were evaluated using the following tests:

- Stride Velocity [m/s] and Stride Length [cm] were obtained from a 10 m walk test using a motion capture system. Each participant performed two trials at their usual walking speed, and the mean value was recorded. These variables provide reliable indicators of mobility, coordination, and lower-limb performance, with longer stride length associated with greater muscular strength and reduced fall risk [20,21].
- Timed Up and Go Test [TUG, seconds] evaluated functional mobility and dynamic balance by timing the participant as they stood up from a chair, walked three meters, turned, and sat down again. Shorter completion times indicate better mobility and lower fall risk [22].
- Functional Reach Test [FRT, cm] measured the maximal distance a participant could reach forward without taking a step, providing a valid measure of dynamic balance and stability limits [23].

All balance tests were administered by the same physiotherapist, who was blinded to group allocation, ensuring consistency and reducing measurement bias.

#### 2.2.3. Secondary Outcome Measures

**Lower-Limb Muscular Power**

Lower-limb muscular power was assessed through three functional performance tests that simulate daily activities and have been widely used in older adult populations: the Chair Stand Test [CST], the Stair Ascend Test [SAT], and the Stair Descend Test [SDT].

The CST measured the time and relative power [W/kg] required to perform five consecutive sit-to-stand repetitions, reflecting lower-limb strength, coordination, and movement velocity. The SAT quantified the time and estimated power required to climb a standardized flight of stairs, representing concentric muscular effort, while the SDT assessed eccentric control and neuromuscular coordination during stair descent. Although these field-based tests are not considered gold-standard methods for power measurement, they are validated, reliable, and clinically relevant indicators of functional lower-limb performance in older adults [24,25]. Their use has been supported as practical alternatives in community and clinical settings, where laboratory-based instruments are not feasible.

Moreover, these tests are strongly correlated with lower-limb strength, mobility, and independence in older adults, and predict key outcomes such as gait speed, fall risk, and activities of daily living [26,27,28].

Each test was performed twice, with the best result recorded. Testing sessions were supervised by a physiotherapist blinded to group allocation to ensure methodological consistency and reproducibility. Together, these measures provided a valid functional representation of muscular power and performance in daily tasks among older adults.

## 3. Intervention

Participants assigned to the CrossFit-adapted group followed a 12-week supervised program consisting of three weekly sessions [Monday, Wednesday, and Friday], each lasting approximately 60 min. A maximum of eight participants per session was allowed to ensure adequate supervision, individualized scaling, and safety. Each session was supervised by a licensed physiotherapist and centered on teaching proper technique for each exercise and how to adjust the level of difficulty.

The CrossFit-adapted program incorporated functional exercises and multi-joint movements that mimicked daily tasks. Movements were selected to progressively challenge strength, coordination, and mobility, while allowing individualized modifications. For example, in the deadlift exercise, weaker participants began with a light kettlebell and gradually progressed, whereas those with higher capacity were encouraged to increase load or complexity while maintaining individualized challenge and safety.

The training structure was standardized across all sessions as follows:

Warm-up [10–15 min]: joint mobility, dynamic stretching, and light cardiovascular activity [e.g., walking or rowing].

Main workout [30–35 min]: multi-joint functional movements emphasizing lower- and upper-limb strength, balance, and mobility, including sit-to-stand variations, loaded carries, step-ups, kettlebell deadlifts, rowing, and stability drills. The exact exercises, sets, and repetitions performed during the program are detailed in Table A2 in Appendix A.

Cool-down [10 min]: static stretching and controlled breathing exercises.

Exercise intensity was self-monitored using the Borg Rating of Perceived Exertion scale, with a target intensity of 5–7 [“moderate to somewhat hard”], which corresponds to approximately 60–75% of individual capacity according to established exercise guidelines for older adults [29]. This range was selected to optimize physiological adaptation while preventing fatigue or overtraining.

The control group maintained their usual lifestyle without structured exercise. They received a brief, single health-education session delivered individually by a licensed physiotherapist, lasting approximately 15 min, covering general recommendations on mobility, physical activity, fall prevention, and healthy aging. No additional sessions or follow-up content were provided during the study period. After completing the intervention, participants in the control group were offered the same CrossFit-adapted program.

Adherence was quantified exclusively through the number of sessions attended, recorded by the supervising physiotherapist throughout the 12-week period for both intention-to-treat and per-protocol analyses.

**Intervention Fidelity**

To ensure consistency and reproducibility of the CrossFit-adapted program, multiple fidelity procedures were implemented throughout the 12-week intervention. All training sessions were delivered and supervised by the same licensed physiotherapist, who had prior experience in functional and high-intensity training for older adults. Before the start of the study, the instructor completed a standardized training protocol outlining the objectives, structure, and scaling criteria of each exercise.

The content and progression of all sessions followed a detailed session template (Table A2 in Appendix A), which specified the warm-up, main workout, and cool-down components, along with the precise execution and modification options for each exercise. To maintain fidelity across participants and sessions, the physiotherapist monitored technique continuously, ensured appropriate scaling according to individual capacity, and maintained target intensity using the Borg Rating of Perceived Exertion scale.

Attendance and session characteristics (exercise selection, load progression, modifications, and perceived exertion) were documented immediately after each session using standardized log sheets. These records allowed verification that the intervention was delivered as intended and that participants received a comparable training stimulus across sessions. No deviations from the planned protocol were reported.

### Statistical Analysis

The sample size was calculated a priori using G*Power 3.1 software [30], considering an effect size of 0.25 [medium], α = 0.05, and a power [1–β] of 0.80 for a repeated-measures ANOVA with two groups and three measurement points. The analysis indicated that a minimum of 54 participants would be required. To account for potential dropouts, 60 participants were recruited [30 per group].

Data were analyzed using SPSS Statistics version 25.0 [IBM Corp., Armonk, NY, USA]. Normality of distribution was verified with the Shapiro–Wilk test, and homogeneity of variances with Levene’s test. Between-group and within-group effects over time were analyzed using repeated-measures ANOVA with Bonferroni-adjusted post hoc comparisons. Effect sizes were calculated using Cohen’s d [31], and interpreted as small [0.2], moderate [0.5], and large [0.8]. Statistical significance was set at *p* < 0.05. All analyses were performed following a per-protocol approach. Only participants who attended ≥85% of sessions and completed both baseline and post-intervention assessments were included in the final analysis. No intention-to-treat [ITT] analysis or imputation of missing data was performed.

## 4. Results

### 4.1. Participant Flow, Adherence and Safety

**Figure 1:**
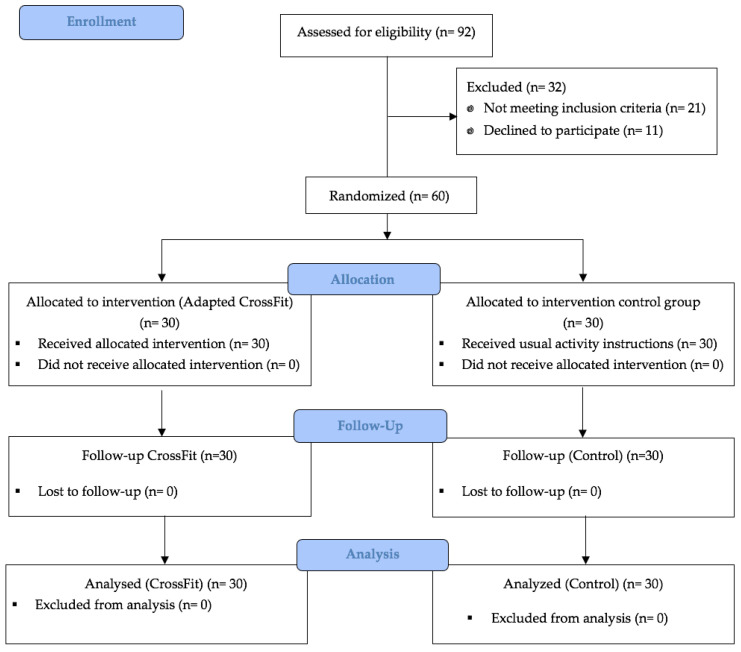
Flowchart Diagram According to CONSORT Statement for the Report of Randomized Controlled Trials.

Figure 1 shows the flow of participants through the study, including screening, eligibility, randomization, and final analysis. All participants completed the intervention and follow-up assessments. Adherence to the intervention was quantified through the number of sessions attended across the 12-week program, with high compliance observed. No adverse events or training-related injuries were reported during the study period.

### 4.2. Baseline Characteristics

A total of 60 older adults [32 women and 28 men] participated in the study. The baseline characteristics of the participants are presented in Table 1. No significant differences were observed between groups regarding age, gender, anthropometric variables, handgrip strength, or physical activity levels [all *p* > 0.05]. This indicates that the CrossFit-adapted [CFA] and control [CON] groups were homogeneous at baseline, enabling valid comparisons of training effects.

### 4.3. Functional Capacity and Balance Outcomes

The analysis revealed significant Group × Time interaction effects for all primary and several secondary outcomes. Specifically, significant interactions were found for the Timed Up and Go [TUG] and Functional Reach Test [FRT] [*p* < 0.001 for both], as well as for gait-related variables including stride velocity and stride length, and for cervical range of motion [all *p* < 0.05]. These results indicate greater improvements in the CrossFit-adapted group compared with the control group across these domains.

Within-group analyses indicated that the CrossFit-adapted group showed significant pre-post improvements in TUG [*p* < 0.001], FRT [*p* < 0.001], stride velocity [*p* < 0.01], stride length [*p* < 0.01], and cervical range of motion [*p* < 0.05], whereas no significant changes were observed in the control group.

Between-group post-intervention comparisons demonstrated significantly greater performance in the CrossFit-adapted group compared with the control group for TUG, FRT, stride velocity, stride length, and cervical range of motion [all *p* < 0.05].

Corresponding results are presented in Table 2 and Table 3.

### 4.4. Lower Limb Muscle Power

Significant Group × Time interaction effects were observed for all lower-limb muscle power outcomes assessed through the Chair Stand Test [CST], Stair Ascend Test [SAT], and Stair Descend Test [SDT] [all *p* < 0.001]. These results indicate a superior improvement pattern in the CrossFit-adapted group compared with the control group over the 12-week period.

Post hoc within-group analyses showed that participants in the CrossFit-adapted group experienced significant increases in lower-limb muscle power across all three tests, with faster completion times and higher estimated power outputs for CST, SAT, and SDT [all *p* < 0.001]. In contrast, no significant pre–post changes were observed in the control group.

Between-group post-intervention comparisons also demonstrated significantly higher muscle power performance in the CrossFit-adapted group than in the control group for CST, SAT, and SDT [all *p* < 0.05], confirming the superiority of the training stimulus.

Overall, these findings suggest that the CrossFit-adapted program effectively improved lower-limb power, an essential component for functional independence and daily mobility among older adults. Detailed results for all muscle power measures are presented in Table 4.

## 5. Discussion

The aim of this study was to examine the effects of a 12-week CrossFit-adapted training program on balance, dynamic stability, and lower-limb muscular power in older adults, and to compare its outcomes with those of a control group that maintained usual daily activities. The results showed significant improvements in all balance and strength-related outcomes in the CrossFit-adapted group, while no significant changes were observed in the control group. These findings suggest that a supervised and progressively adapted CrossFit-based program can effectively enhance physical function and neuromuscular performance in community-dwelling older adults [18,32]. These results are particularly relevant given the aging population and the growing need for interventions that improve mobility, reduce fall risk, and enhance overall physical functioning in older adults.

The findings of this study indicate that a CrossFit-adapted program can serve as an effective and feasible multimodal training approach to enhance balance, mobility, and lower-limb power in older adults. These results are consistent with previous evidence showing that various structured exercise modalities—including strength, functional, and multicomponent training—can improve physical performance when properly prescribed and supervised [29,33,34]. The CrossFit-adapted program therefore represents one practical and motivating option within the wide range of effective exercise strategies available for this population. This is in line with the findings of Müller et al. [35], who found that both traditional strength training and power training, when combined with high-intensity interval training [HIIT], equally improved functional capacity, cardiorespiratory fitness, and body composition in older men. The combination of functional exercises with strength training, as demonstrated in our study, further supports the idea that a varied and dynamic training approach can effectively address the multifaceted needs of older adults.

Our study’s findings also align with the work of Rivas-Campo et al. [36], who observed improvements in functional capacity, balance, and daily activities in older adults participating in a high-intensity functional training [HIFT] program. The significant improvements observed in balance and gait stability in our participants suggest that a CrossFit-adapted program, which incorporates exercises that challenge both strength and coordination, can be an effective means of enhancing balance and reducing the risk of falls in older adults. Moreover, the improvement in activities of daily living, observed in the present study, highlights the importance of integrating functional training that mimics real-life movements to maintain independence in older adults.

However, despite the clear benefits of the CrossFit-adapted program, the results were not without nuance. As observed by Wong et al. [37], strength training is a crucial component in promoting resilience among older adults, and while the CrossFit-adapted program showed positive effects on strength, further research is needed to examine its long-term impact on resilience. Additionally, while our study provides evidence for the effectiveness of CrossFit for enhancing physical fitness, more research is needed to explore its potential cognitive benefits, especially since some studies, like those of Rivas-Campo et al. [36], have suggested that functional training can also improve cognitive functions in older adults. This is an area that warrants further investigation, particularly in terms of whether such physical interventions may have dual benefits for both physical and cognitive health.

Interestingly, our results showed significant improvements in functional performance tests such as the sit-to-stand and Timed Up and Go [TUG] tests, which reflect global neuromuscular function and mobility. These findings suggest that the CrossFit-adapted intervention effectively enhanced lower-limb strength, coordination, and balance, contributing to better functional capacity in daily tasks. Similar results were reported by Rivas-Campo et al. [36], who also observed no improvements in this parameter despite gains in other physical domains. Future research should include targeted upper body exercises to provide a more comprehensive and balanced training approach for older adults.

Another key consideration is adherence to the program. Our study highlights the importance of maintaining engagement and consistency in exercise programs for older adults. In our sample, participants in the CrossFit-adapted group attended 94% of the planned sessions, demonstrating excellent adherence to the intervention. This high level of adherence may have contributed to the observed improvements in functional outcomes, underscoring the relevance of structured, supervised programs to maintain the long-term participation in physical activity among older adults. Future research should explore strategies to enhance adherence to such programs, particularly in ensuring that the exercises are enjoyable and sustainable for participants.

One limitation of this study was the absence of long-term follow-up to evaluate the sustainability of the improvements observed after the CrossFit-adapted program. Although significant gains were detected in strength, balance, and functional performance, it remains uncertain whether these effects would persist without continued training. Future research should address this issue by including follow-up assessments to examine the durability of the intervention’s outcomes over time.

Another relevant consideration concerns the limited generalizability of the findings. The sample consisted of relatively healthy older adults who were able to complete the training protocol without major physical or cognitive impairments. As a result, the applicability of these results to individuals with greater functional limitations, frailty, or cognitive decline remains uncertain. Expanding future investigations to include more diverse populations would provide a clearer understanding of the feasibility and adaptability of CrossFit-based interventions across different functional levels.

Finally, although the present study effectively evaluated several physical outcomes, it did not include measures related to psychological or social dimensions. Prior evidence emphasizes the influence of social interaction, motivation, and psychological well-being on adherence and overall benefits of physical activity in older adults [36]. Including these aspects in future research would contribute to a more comprehensive understanding of the broader impact of CrossFit-adapted programs on health and quality of life.

An additional aspect to consider when interpreting our findings is the unusually high level of self-reported physical activity observed at baseline, which clearly exceeds current WHO recommendations. Although physical activity was not a variable under direct investigation in this study, this characteristic of the sample is relevant for contextualizing our results. Similar magnitudes of weekly activity have been described in selected cohorts of community-dwelling older adults when detailed self-report instruments are used. For example, Wingood et al. [38] documented approximately 10 h of weekly physical activity in volunteer samples, suggesting that highly active individuals may be more inclined to participate in structured, supervised exercise programs such as ours. Furthermore, self-reported measures are known to overestimate moderate-to-vigorous activity compared with objective monitoring devices, particularly in older populations [39,40]. Therefore, the elevated baseline values observed in our sample likely reflect both a particularly active and health-motivated cohort, as well as the inherent overestimation associated with self-report methods. These factors should be considered when interpreting and generalizing the present findings.

This study presents several limitations that should be acknowledged. First, the sample size, although adequate for detecting medium effects, limits the generalizability of the findings and may not capture more subtle subgroup differences. Second, the 12-week duration provides insight into short-term adaptations but does not allow conclusions about the long-term sustainability of the improvements observed. Third, the lack of follow-up prevents determining whether gains in balance, mobility, and muscle power persist beyond the intervention period. Future studies should incorporate extended follow-up assessments, larger and more diverse samples, and comparisons with other evidence-based exercise modalities to better understand long-term adherence, durability of effects, and the applicability of CrossFit-adapted programs across different functional and clinical profiles of older adults.

Clinical Application

Although the intervention yielded statistically significant improvements in functional performance measures, it is essential to interpret these changes in terms of clinical relevance, particularly for outcomes directly associated with autonomy and the safe execution of daily activities in older adults. Several of the improvements observed in this study exceeded established minimal clinically important difference [MCID] thresholds. For example, changes in the Timed Up and Go [TUG] test surpassed the MCID range of 0.8–1.4 s reported for older adults [41], and improvements in the Functional Reach Test [FRT] exceeded the commonly accepted threshold of approximately 5 cm [39]. Likewise, the increase in gait speed observed in the CrossFit-adapted group exceeded the meaningful change of ≥0.10 m/s that reflects clinically relevant mobility gains [42]. Furthermore, enhancements in lower-limb muscle power are consistent with clinically meaningful improvements described in the literature, given the strong association between chair-stand performance, functional mobility, and lower-limb strength in older adults [43,44,45]. Taken together, these findings indicate that the magnitude of change achieved in our intervention is not only statistically significant but also clinically meaningful, with clear implications for maintaining independence in community-dwelling older adults.

In terms of clinical relevance, the magnitude of change observed in the Timed Up and Go (TUG) test is particularly meaningful. The 1.2 s reduction achieved by the CrossFit-adapted group exceeds the minimal clinically important difference (MCID) reported for community-dwelling older adults, which ranges from 0.8 to 1.4 s. Improvements of this magnitude have been associated with better functional mobility and a lower likelihood of future falls, as slower TUG performance is a well-established predictor of fall risk. Therefore, the observed improvement reflects not only statistical significance but also a clinically meaningful enhancement in functional independence and safety during daily mobility.

## 6. Conclusions

In conclusion, the 12-week CrossFit-adapted training program produced significant improvements in balance, dynamic stability, and lower-limb muscular power in older adults. These findings support the use of structured, supervised, and scalable functional exercise programs based on CrossFit methodology as a safe and effective approach to enhance physical function and independence in this population.

Importantly, the high adherence observed throughout the intervention highlights adaptability to various functional levels of older adults and its potential to improve multiple domains of physical health. Future studies should incorporate quality-of-life and psychosocial measures, longer follow-up periods, and comparisons with other exercise modalities to better determine the long-term benefits and broader impact of CrossFit-based interventions in aging populations.

## Figures and Tables

**Table 1:** Baseline characteristics of participants by group.

Variable	CFA [n = 30] (95% CI)	CON [n = 30] (95% CI)	*p*-Value
Age, years	72.8 ± 5.1 (70.5–75.1)	72.5 ± 4.9 (70.3–74.7)	0.74
Gender Male	8(26.66%)	11 (36.66%)	0.57
Female	22 (73.34%)	19 (63.34%)	
Height, cm	165.9 ± 7.2 (163–169)	169.0 ± 6.8 (166–172)	0.31
Body mass, kg	70.3 ± 8.5 (67–74)	74.4 ± 9.0 (70–78)	0.28
BMI, kg/m^2^	25.5 ± 2.1 (24.6–26.4)	26.1 ± 2.2 (25.1–27.1)	0.34
Muscle mass, kg	26.7 ± 3.5 (25–28)	27.7 ± 3.7 (26–29)	0.40
Handgrip strength, kg	30.1 ± 5.0 (28–32)	28.6 ± 5.2 (26–31)	0.29
Physical activity, h/week	15.6 ± 3.8 (14–17)	14.4 ± 3.9 (13–16)	0.37

BMI [body mass index]; CFA [CrossFit-adapted]; CON [Control].

**Table 2:** Balance outcomes before and after the 12-week CrossFit-adapted program.

Variable	CFA Pre	CFA Post (95% IC)	CON Pre	CON Post (95% IC)	*p*-Value [Group × Time]	Cohen’s d
ROM, s	12.7 ± 2.1	21.2 ± 2.9 (20.12–22.28)	15.7 ± 2.3	12.3 ± 2.5 (11.37–13.23)	<0.001	2.50
Stride velocity, m/s	1.36 ± 0.08	1.46 ± 0.09 (1.43–1.49)	1.35 ± 0.09	1.34 ± 0.10 (1.30–1.38)	0.004	1.18
Stride length, cm	141.3 ± 5.5	146.0 ± 6.1 (143.72–148.28)	140.3 ± 5.7	138.4 ± 6.2 (136.09–140.71)	0.044	0.80
TUG, s	9.83 ± 1.3	8.74 ± 1.1 (8.33–9.15)	9.85 ± 1.2	9.64 ± 1.2 (9.19–10.09)	0.002	0.86
FRT, cm	30.5 ± 3.2	36.0 ± 3.8 (34.58–37.42)	32.7 ± 3.6	32.0 ± 3.9 (30.54–33.46)	<0.001	1.51

Values are presented as mean ± SD. Post values include 95% CI. *p*-values represent the Group × Time interaction from the two-way mixed ANOVA, indicating differences in pre–post changes between groups. Within-group comparisons [pre vs. post] were also examined and showed significant improvements only in the CrossFit-adapted group [CFA]. CFA = CrossFit-adapted group; CON = Control group; ROM = Romberg test; TUG = Timed Up and Go test; FRT = Functional Reach Test. Significance levels: *p* < 0.05; *p* < 0.01; *p* < 0.001.

**Table 3:** Repeated measures ANOVA results for balance and functional mobility variables.

Variable	Main Effect of Time [*p*]	Main Effect of Group [*p*]	Group × Time Interaction [*p*]
ROM [s]	<0.001	<0.001	<0.001
Stride velocity [m/s]	0.002	0.205	0.004
Stride length [cm]	0.022	0.591	0.044
TUG [s]	<0.001	0.229	0.002
FRT [cm]	<0.001	<0.001	<0.001

ROM [Romberg test]; TUG [Timed Up and Go test]; FRT [Functional Reach Test].

**Table 4:** Lower limb muscle power outcomes before and after the 12-week CrossFit-adapted program.

Variable	CFA Pre	CFA Post (95% IC)	CON Pre	CON Post (95% IC)	*p*-Value	Cohen’s d
CST, s	12.8 ± 1.9	9.8 ± 1.6 (9.20–10.40)	11.7 ± 1.8	11.6 ± 1.9 (10.89–12.31)	<0.001	1.72
CST, W/kg	8.4 ± 1.6	10.0 ± 1.8 (9.33–10.67)	9.1 ± 1.5	9.2 ± 1.6 (8.60–9.80)	<0.001	0.92
SAT, s	5.4 ± 0.7	4.5 ± 0.6 (4.28–4.72)	5.2 ± 0.8	5.0 ± 0.8 (4.70–5.30)	<0.001	1.37
SAT, W/kg	2.6 ± 0.3	3.0 ± 0.3 (2.89–3.11)	2.6 ± 0.3	2.7 ± 0.3 (2.59–2.81)	<0.001	1.33
SDT, s	5.0 ± 0.6	3.8 ± 0.5 (3.61–3.99)	4.5 ± 0.6	4.3 ± 0.6 (4.08–4.52)	<0.001	2.09
SDT, W/kg	2.8 ± 0.3	3.5 ± 0.3 (3.39–3.61)	3.1 ± 0.3	3.2 ± 0.3 (3.09–3.31)	<0.001	2.33

CFA = CrossFit-adapted group; CON = Control group; CST = Chair Stand Test; SAT = Stair Ascend Test; SDT = Stair Descend Test. Values are mean ± SD; Post values include 95% CI.

## Data Availability

The data presented in this study are available on request from the corresponding author. The data are not publicly available due to privacy and ethical restrictions.

## Abbreviations

The following abbreviations are used in this manuscript:

BIA	Bioelectrical impedance analysis
BMI	Body mass index
CST	Chair Stand Test
CON	Control
FRT	Functional Reach Test
HIFT	High-intensity functional training
HIIT	High-intensity interval training
MMSE	Mini-Mental State Examination
ROM	Romberg test
SAT	Stair Ascend Test
SDT	Stair Descend Test
CFA	CrossFit-adapted
TST	Traditional strength training
TUG	Time up and go
VO_2_max	Maximum oxygen volume

## Appendix A

**Table A1:** Baseline Variables and Data Source.

Variable	Description/Units	Data Source
Age	Years	Hospital clinical–research database
Sex	Male/Female	Hospital clinical–research database
Height	cm	Hospital clinical–research database
Weight	kg	Hospital clinical–research database
Body Mass Index [BMI]	kg/m^2^	Hospital clinical–research database
Number of chronic conditions [*it appears in your baseline*]	Count of diagnosed chronic diseases	Hospital clinical–research database
Cognitive screening [MMSE]	Mini-Mental State Examination (0–30)	Direct baseline assessment by physiotherapists
Self-reported physical activity	Minutes/week or categorical scale	Direct baseline assessment
Handgrip strength	kg, dynamometer test [best of 2 attempts]	Direct baseline assessment
Timed Up and Go [TUG]	Seconds	Direct baseline assessment
Functional Reach Test [FRT]	cm	Direct baseline assessment
Gait speed/Stride velocity	m/s	Direct baseline assessment
Romberg test	Seconds, eyes open	Direct baseline assessment
Balance and mobility battery [according to protocol]	As described in Section 2.2	Direct baseline assessment

**Table A2:** Structure of the CrossFit-Adapted Training Sessions.

Exercise	Modality	Sets	Repetitions/Time	Notes [Scaling Options]
Sit-to-Stand/Box Squat	Strength/Functional	3	8–12 reps	Box height adapted to ability
Seated/Standing Shoulder Press	Strength	3	8–10 reps	Adjustable dumbbells [1–6 kg]
Deadlift with Kettlebell	Strength	3	6–10 reps	4–12 kg depending on capacity
Step-ups	Functional	3	10 reps each leg	Step height individualized
Farmer’s Carry	Conditioning	3	20–30 m	Light–moderate load
Marching in Place	Balance/Warm-up	2	30–40 s	Support if needed
“AMRAP 6 min” [Sit-to-Stand, Step-ups, Carry]	Circuit	1	6 min continuous	Perceived exertion target 5–7
Cool-down	Flexibility	–	5 min	Stretching of major muscle groups

## References

[1] Baert V., Gorus E., Calleeuw K., De Backer W., Bautmans I. (2016). An Administrator’s Perspective on the Organization of Physical Activity for Older Adults in Long-Term Care Facilities. J. Am. Med. Dir. Assoc..

[2] https://library.crossfit.com/free/pdf/CFJ_56-07_Understanding.pdf.

[3] Mangine G.T., Stratton M.T., Almeda C.G., Roberts M.D., Esmat T.A., VanDusseldorp T.A., Feito Y. (2020). Physiological differences between advanced CrossFit athletes, recreational CrossFit participants, and physically-active adults. PLoS ONE.

[4] Fisher J., Steele J., McKinnon P., McKinnon S. (2014). Strength Gains as a Result of Brief, Infrequent Resistance Exercise in Older Adults. J. Sports Med. Hindawi Publ. Corp..

[5] Pereira M.J., Mendes R., Mendes R.S., Martins F., Gomes R., Gama J., Dias G., Castro M.A. (2022). Benefits of Pilates in the Elderly Population: A Systematic Review and Meta-Analysis. Eur. J. Investig. Health Psychol. Educ..

[6] Stødle I.V., Debesay J., Pajalic Z., Lid I.M., Bergland A. (2019). The experience of motivation and adherence to group-based exercise of Norwegians aged 80 and more: A qualitative study. Arch. Public Health.

[7] Franco M.R., Tong A., Howard K., Sherrington C., Ferreira P.H., Pinto R.Z., Ferreira M.L. (2015). Older people’s perspectives on participation in physical activity: A systematic review and thematic synthesis of qualitative literature. Br. J. Sports Med..

[8] Moxley E.W., Smith D., Quinn L., Park C. (2018). Relationships Between Glycemic Control and Cardiovascular Fitness. Biol. Res. Nurs..

[9] Barreto A.C., Medeiros A.P., Araujo G.d.S., Vale R., Vianna J.M., Alkimin R., Serra R., Leitão L., Reis V.M., da Silva Novaes J. (2023). Heart rate variability and blood pressure during and after three CrossFit^®^ sessions. Retos.

[10] Marston K.J., Peiffer J.J., Rainey-Smith S.R., Gordon N., Teo S.Y., Laws S.M., Sohrabi H.R., Martins R.N., Brown B.M. (2019). Resistance training enhances delayed memory in healthy middle-aged and older adults: A randomised controlled trial. J. Sci. Med. Sport.

[11] Jiménez-García J.D., Martínez-Amat A., De la Torre-Cruz M.J., Fábrega-Cuadros R., Cruz-Díaz D., Aibar-Almazán A., Achalandabaso-Ochoa A., Hita-Contreras F. (2019). Suspension Training HIIT Improves Gait Speed, Strength and Quality of Life in Older Adults. Int. J. Sports Med..

[12] Costa A., Silva P.L.G., Fontes P.A. (2025). O impacto do treinamento de CrossFit e treinamento funcional na autonomia funcional de idosos—Uma revisão integrativa. Res. Soc. Dev..

[13] Niyazi A., Mir E., Ghasemi Kahrizsangi N., Mohammad Rahimi N., Fazolahzade Mousavi R., Setayesh S., Nejatian Hoseinpour A., Mohammad Rahimi F., Mohammad Rahimi G.R. (2024). The effect of functional exercise program on physical functioning in older adults aged 60 years or more: A systematic review and meta-analysis of randomized controlled trials. Geriatr. Nur..

[14] Gean R.P., Martin R.D., Cassat M., Mears S.C. (2020). A Systematic Review and Meta-analysis of Injury in Crossfit. J. Surg. Orthop. Adv..

[15] Mehrab M., Wagner R.K., Vuurberg G., Gouttebarge V., de Vos R.-J., Mathijssen N.M.C. (2023). Risk Factors for Musculoskeletal Injury in CrossFit: A Systematic Review. Int. J. Sports Med..

[16] Ageing and Health. https://www.who.int/news-room/fact-sheets/detail/ageing-and-health.

[17] Bull F.C., Al-Ansari S.S., Biddle S., Borodulin K., Buman M.P., Cardon G., Carty C., Chaput J.-P., Chastin S., Chou R. (2020). World Health Organization 2020 guidelines on physical activity and sedentary behaviour. Br. J. Sports Med..

[18] Lesinski M., Hortobágyi T., Muehlbauer T., Gollhofer A., Granacher U. (2015). Effects of Balance Training on Balance Performance in Healthy Older Adults: A Systematic Review and Meta-analysis. Sports Med..

[19] Granacher U., Gollhofer A., Hortobágyi T., Kressig R.W., Muehlbauer T. (2013). The importance of trunk muscle strength for balance, functional performance, and fall prevention in seniors: A systematic review. Sports Med..

[20] Lord S.R., Lloyd D.G., Li S.K. (1996). Sensori-motor function, gait patterns and falls in community-dwelling women. Age Ageing.

[21] Hollman J.H., McDade E.M., Petersen R.C. (2011). Normative spatiotemporal gait parameters in older adults. Gait Posture.

[22] Podsiadlo D., Richardson S. (1991). The timed “Up & Go”: A test of basic functional mobility for frail elderly persons. J. Am. Geriatr. Soc..

[23] Duncan P.W., Weiner D.K., Chandler J., Studenski S. (1990). Functional reach: A new clinical measure of balance. J. Gerontol..

[24] Bean J.F., Kiely D.K., Herman S., Leveille S.G., Mizer K., Frontera W.R., Fielding R.A. (2002). The relationship between leg power and physical performance in mobility-limited older people. J. Am. Geriatr. Soc..

[25] Alcazar J., Losa-Reyna J., Rodriguez-Lopez C., Alfaro-Acha A., Rodriguez-Mañas L., Ara I., García-García F.J., Alegre L.M. (2018). The sit-to-stand muscle power test: An easy, inexpensive and portable procedure to assess muscle power in older people. Exp. Gerontol..

[26] Csuka M., McCarty D.J. (1985). Simple method for measurement of lower extremity muscle strength. Am. J. Med..

[27] Reid K.F., Fielding R.A. (2012). Skeletal muscle power: A critical determinant of physical functioning in older adults. Exerc. Sport Sci. Rev..

[28] Alcazar J., Kamper R.S., Aagaard P., Haddock B., Prescott E., Ara I., Suetta C. (2020). Relation between leg extension power and 30-s sit-to-stand muscle power in older adults: Validation and translation to functional performance. Sci. Rep..

[29] Nelson M.E., Rejeski W.J., Blair S.N., Duncan P.W., Judge J.O., King A.C., Macera C.A., Castaneda-Sceppa C. (2007). Physical activity and public health in older adults: Recommendation from the American College of Sports Medicine and the American Heart Association. Med. Sci. Sports Exerc..

[30] Kang H. (2021). Sample size determination and power analysis using the G*Power software. J. Educ. Eval. Health Prof..

[31] Cohen J. (1988). Statistical Power Analysis for the Behavioral Sciences.

[32] Chase J.-A.D., Phillips L.J., Brown M. (2017). Physical Activity Intervention Effects on Physical Function Among Community-Dwelling Older Adults: A Systematic Review and Meta-Analysis. J. Aging Phys. Act..

[33] Cadore E.L., Izquierdo M. (2013). How to simultaneously optimize muscle strength, power, functional capacity, and cardiovascular gains in the elderly: An update. Age.

[34] Fragala M.S., Cadore E.L., Dorgo S., Izquierdo M., Kraemer W.J., Peterson M.D., Ryan E.D. (2019). Resistance Training for Older Adults: Position Statement From the National Strength and Conditioning Association. J. Strength Cond. Res..

[35] Müller D.C., Izquierdo M., Boeno F.P., Aagaard P., Teodoro J.L., Grazioli R., Radaelli R., Bayer H., Neske R., Pinto R.S. (2020). Adaptations in mechanical muscle function, muscle morphology, and aerobic power to high-intensity endurance training combined with either traditional or power strength training in older adults: A randomized clinical trial. Eur. J. Appl. Physiol..

[36] Rivas-Campo Y., Aibar-Almazán A., Afanador-Restrepo D.F., García-Garro P.A., Vega-Ávila G.C., Rodríguez-López C., Castellote-Caballero Y., Carcelén-Fraile M.D.C., Lavilla-Lerma M.L. (2023). Effects of High-Intensity Functional Training (HIFT) on the Functional Capacity, Frailty, and Physical Condition of Older Adults with Mild Cognitive Impairment: A Blind Randomized Controlled Clinical Trial. Life.

[37] Wong M.Y.C., Zhang C.-Q., Zhao Y., Hu C., Ou K. (2024). Effectiveness of resistance training on resilience in Hong Kong Chinese older adults. Cogent Psychol..

[38] Wingood M., Bonnell L., LaCroix A.Z., Rosenberg D., Walker R., Bellettiere J., Greenwood-Hickman M.A., Wing D., Gell N. (2022). Community-Dwelling Older Adults and Physical Activity Recommendations: Patterns of Aerobic, Strengthening, and Balance Activities. J. Aging Phys. Act..

[39] Prince S.A., Adamo K.B., Hamel M.E., Hardt J., Connor Gorber S., Tremblay M. (2008). A comparison of direct versus self-report measures for assessing physical activity in adults: A systematic review. Int. J. Behav. Nutr. Phys. Act..

[40] Herbolsheimer F., Riepe M.W., Peter R. (2018). Cognitive function and the agreement between self-reported and accelerometer-accessed physical activity. BMC Geriatr..

[41] Wright A.A., Cook C.E., Baxter G.D., Dockerty J.D., Abbott J.H. (2011). A comparison of 3 methodological approaches to defining major clinically important improvement of 4 performance measures in patients with hip osteoarthritis. J. Orthop. Sports Phys. Ther..

[42] Perera S., Mody S.H., Woodman R.C., Studenski S.A. (2006). Meaningful change and responsiveness in common physical performance measures in older adults. J. Am. Geriatr. Soc..

[43] Bohannon R.W., Glenney S.S. (2014). Minimal clinically important difference for change in comfortable gait speed of adults with pathology: A systematic review. J. Eval. Clin. Pract..

[44] Jones C.J., Rikli R.E., Beam W.C. (1999). A 30-s chair-stand test as a measure of lower body strength in community-residing older adults. Res. Q. Exerc. Sport.

[45] Rikli R.E., Jones C.J. (1999). Development and Validation of a Functional Fitness Test for Community-Residing Older Adults. J. Aging Phys. Act..

